# Effect of educational intervention on improvement of physical activities of middle-aged women

**DOI:** 10.1186/s12905-021-01494-z

**Published:** 2021-10-09

**Authors:** Tayebeh Rakhshani, Zahra Khiyali, Fatemeh Masrurpour, Ali Khani Jeihooni

**Affiliations:** 1 Nutrition Research Center, Department of Public Health, School of Public Health, Shiraz University of Medical Sciences, Shiraz, Iran; 2 Department of Public Health, School of Public Health, Fasa University of Medical Sciences, Fasa, Iran

**Keywords:** Educational intervention, Self-efficacy, Physical activity, Middle-aged women

## Abstract

**Background:**

Regular physical activity has important health benefits for women. The present study aimed to investigate the effect of theory-based educational interventions on the improvement of physical activities of middle-aged women.

**Methods:**

This experimental study was conducted on 160 middle-aged women referred to Ahwaz health centers Iran in 2017 who were selected through the cluster sampling method. They were randomly divided into two groups: intervention (n = 80) and control (n = 80). The educational intervention for the experimental group consisted of 4 consultation and 4 follow-up sessions. The study outcomes included physical activity change, self-efficacy, decision-making balance, and the International Physical Activity Questionnaire, which were measured before the intervention and 2 months after that. The data were analyzed using the SPSS19 software as well as the Chi-square, independent t-test and paired t-test.

**Results:**

After the intervention, the mean scores of preparedness for change, decision-making balance, perceived self-efficacy, pre-thinking, thinking, preparation, practice and maintenance were significantly different in both the experimental and control groups (*P* value < 0.05). The t-test showed that after the educational intervention, the mean score of physical activity was significantly higher in the experimental group than in the control group (726.3 ± 201.6 and 339.8 ± 90.2, respectively) (*P* value < 0.001).

**Conclusion:**

The findings of this study showed that the theory-based educational intervention was effective on the improvement of the middle-aged women’s physical activities. Therefore, it is suggested that a theory-based educational program be designed and implemented in order to increase the physical activities of this group.

## Background

As defined by the World Health Organization (WHO), physical activity refers to any movement that is generated by the movement of skeletal muscles and is associated with energy consumption [1]. The Ministry of Health has divided physical activities into three types based on severity: light, moderate and vigorous. Light physical activities involve usual activities that are done in daily life and require little effort but need daily energy. Moderate physical activities are the activities that increase the number of breathing and heart rate, but the person is able to continue to talk. Vigorous physical activities are those that greatly increase heart rate and respiration, and the person is no longer able to speak [2]. The WHO recommendation on the amount of physical activities required for people aged 18–65 years is to do at least 150 min of moderate physical activities per week, or 75 min of vigorous physical activities weekly, or a balanced combination of moderate and vigorous ones [3].

The global estimate of the rate of immobility among the people over the age of 18 was about 23% in 2010. It was 20% among men and 27% among women. However, immobility is seen in the Eastern Mediterranean region more than any other area in the world, such that more than one-third of the men and almost half of the women in this region are physically inactive [4]. The prevalence of immobility in urban and rural areas are respectively76.3% and 58.8% among Iranian men and women in the age group of 15–64 years, and the total rate of immobility in this age group is 67.5%. Some studies carried out in Iran reported higher rates of immobility [5, 6]. Inactiveness and inadequate physical activities also lead to increased chronic diseases, especially cardiovascular ones, diabetes and osteoporosis [7–9]. All countries in the world are addressing all community groups in the pursuit of health, but among these groups, women, especially middle-aged ones, are of particular importance because they are in conditions that play important roles both in terms of occupational and social responsibilities, and emotions in the center of families. Thus, helping to improve the general health of women in this age group is a kind of help and service to families and societies [10].

Considering the fact that, according to the Department of Middle-aged Health, providing the conditions for the enjoyment of women and men of 30–59 years of age from comprehensive health care services in the country's primary health care system includes lifestyle improvement, fertility and public health promotion through risk factors control, and prevention and early diagnosis of diseases, and also considers the ability of this age group to self-care by relying on self-efficacy in order to promote different dimensions of health and increase their quality of life, it is necessary to take into account the appropriate interventions to enhance the lifestyle of this age group. Lifestyle improvement is the most important strategy in this program, and physical activity is one of its key issues [11]. Choosing appropriate, low-cost, and effective educational methods to change lifestyles and also choosing suitable social settings for access to community need to be investigated. Several studies have identified schools, workplaces, religious sites and health centers as places to facilitate massive behavioral changes in the community [12]. From the perspective of public health education, the creation of a model and the promotion of regular physical activities of women can form the habit of doing regular physical activities in people and promote their health. Besides, a better quality of life can be predicted for their future by motivating the women, informing them and changing their attitudes [13].

One of the models that illustrates regular physical activity behavior behaviors is the Stages of Change Model introduced in the early 1980s by Prochaska & DiClemente to abandon addictive behaviors such as smoking and alcohol consumption [14]. In this model, it is assumed that individuals can be prepared for change at different stages and therefore, they will pass through a set of stages to change their behavior, including: pre-thinking, thinking and intention, preparation, practice and maintenance. Pre- thinking refers to a stage in which a person has not yet thought about changing or adopting a behavior at least for the next six months. At the stage of thinking, the person really thinks of changing behavior during the next six months, but still is not fully prepared to take action. In the preparation stage, the individual thinks seriously about a change in behavior and intends to make a change in the near future (usually next month) and provides the basis for the onset of the behavior. The practice stage is the one in which a person has made the right changes in his/her lifestyle during the last six months. As performance is visible, behavioral change is often said to be performance. In the maintenance stage, a longer period of establishment and strength of behavioral change can be seen. It is for more than six months, but to maintain it, we need to be vigilant and active [15, 16]. Regarding the role of the stages of change model in persuading people to do physical activities, this study aimed to determine the effect of the theory-based educational intervention on the improvement of physical activities of middle-aged women referring to Ahwaz health centers, Iran.

## Methods

This experimental study was conducted in 2017 on the middle-aged women referring to Ahwaz health centers, Iran. The inclusion criteria were as follows: women of 30–59 years of age, being able to read and write, not having chronic diseases such as cardiovascular or respiratory diseases or those that cause physical activities to be banned, not having mobility prohibition (able to move, participate in educational programs, and recommendations for physical activities), lack of pregnancy, lack of specific diseases, lack of a history of accidents resulted in a mental and physical problem during the past month (traffic accident, death of a family member, etc.), and willingness to participate in the research project. The exclusion criteria included the reluctance to participate in the study at any time and not attending various stages of the research (pre-education and post-education assessments, and educational sessions).

In order to determine the sample size and considering the limited statistical population (160 people), the following sampling formula was considered.

$$n = \frac{{\left( {Z_{{1 - \frac{\alpha }{2}}} + Z_{1 - \beta } } \right)^{2} \left( {\delta_{1}^{2} + \delta_{2}^{2} } \right)}}{{\left( {\mu_{1} - \mu_{2} } \right)^{2} }}$$

We used the two-stage cluster sampling method. Firstly, Ahwaz city was divided into 4 geographical regions with approximately equal populations of middle-aged women. Two centers (intervention and control groups) were selected in each region) total 8 centers). Then, each center was referred and based on the inclusion criteria, a list of middle-aged women was prepared from among the household files in the center, and 20 individuals were selected from a simple random selection (in total 160 individual).

We used cluster sampling method based other studies [17–20].

### Educational intervention

The intervention program was conducted for a 2-month period for the intervention group. It consisted of 4 face-to-face consultation sessions, each for 15 minutes in a month, and 4 follow-up sessions (weeks 5- 8) after the consultation sessions for the intervention group. The first consultation session included the completion of a questionnaire for each individual to determine their health status. Then, the researcher, with the help of a training center specialist and a physical education instructor, delivered a speech on physical activity and highlighted its importance, and provided a basis for preparing the participants to change in order to do physical activities. The second session involved a group discussion between the participants in the study and expressing their views on whether physical activity was beneficial or not, so that each participant would reach a decision-making balance and perceived self-efficacy. In the event of a gap in the decision of each participant, the researcher and the psychologist of the center explained and advised them on how to increase their will. The participants were also guided to outline their goals to have physical activities and specify their direction. In the third consultation session, the researcher evaluated the levels of the participants’ thinking and preparation to change by displaying educational videos. The fourth session focused on reaching the goals of the previous three sessions. During the weeks 5-8, the researcher reviewed the extent of the participants’ progress in physical activity and re-evaluated the level of their activities as well as the stage of change. The researcher also encouraged them and tried to find out the reasons for their failure. At the end of the 8^th^ week, the researcher completed the questionnaire on the level of physical activity and the stages of change for the intervention and control groups. It should be noted that after the end of the intervention, the control group was given some sports and health pamphlets.

The data collection tools in this study were a checklist of the women’s demographic information (including their job, spouse's job, education, spouse’s education and income) and the standard questionnaire on physical activity. The questionnaire consisted of two parts. The first part was based on the change structures of the participants’ physical activities. This part comprised of 7 sections: preparation to change (questions 1-5), decision-making balance [6–10], perceived self-efficacy [11–15], pre-thinking [16–20], thinking [21–24], preparation [25–28], practice [29–32] and maintenance [33–36]. The pre-thinking stage is the stage in which people are inactive and do not intend to start regular physical activities in the next six months. The stage of thinking is the one in which people are inactive and are about to begin regular physical activities in the next six months. At the preparation stage, the individuals have irregular physical activities and do them fewer than three times a week and 30 minutes each time. The practice stage is the one in which the individuals have regular physical activities for less than six months. At the maintenance stage, the people regularly exercise for more than six months. The second part of the questionnaire was the short form of the International Physical Activity Questionnaire, which determined the physical activities of the research samples per week based on MET-min/week. Metabolic Equivalent of Task (MET) is a unit used to estimate energy consumption in physical activities. If an individual’s MET is equal to one, it means s/he is inactive. In case the MET is higher than one and less than three, there is low level of physical activity. If the MET is greater than or equal to three and less than six, the intensity of physical activity is moderate, and if the MET is greater than 6, the intensity of physical activity is high. To calculate the intensity of activities, the MET value of each activity is multiplied by the time spent in one day or within a week. This questionnaire was translated by experts and its Cronbach's alpha coefficient was 0.72% based on a pilot study [15]. The Kappa coefficient on the validity of the stages of change questionnaire was also obtained to be 76 by Ghahremani et al. in a study aimed at enhancing physical activities [16].

This study was approved by the Ethics Committee of Shiraz University of Medical Sciences. To describe the data, mean, standard deviation and frequency were used. Besides, to determine the level of consistency between the research samples in both the intervention and control groups, the variables such as age, education level, marital status, place of residence, spouse's education, spouse's occupation and type of housing were used. The Chi-square test was also applied. To examine the effect of education, the Independent T-test test and paired T-test were used as well. The data analysis was performed using the SPSS 19 software and the significance level was considered to be 0.05 in all tests.

## Results

The demographic characteristics of the experimental and control groups are shown in Table 1. The mean and standard deviation of the age of the participants in the intervention and control groups were 38.55 ± 6.82 and 40.06 ± 6.41, respectively. The independent t-test was used to determine the difference between the intervention and control groups in terms of age, and the results showed that there was no significant difference between the two groups in terms of age (*P* value = 0.15). The Chi-square test was also used to determine the difference between the intervention and control groups in terms of job, education level, economic status, spouse’s job and spouse’s education. The test showed that there was no significant difference between the two groups in terms of occupation, education status, income and spouse’s education (Table 1).

**Table 1:** Demographic characteristics of the study participants

Variable	Group				*P* value
	Control		Intervention		
	Number	Percent	Number	Percent	
Type of occupation					
Housewife	52	65	50	62.50	0.22
Employee	12	15	8	10	
Other (retired, etc.)	16	20	22	27.50	
Education level					
Lower than diploma	36	45	36	45	0.25
Associate degree	27	33.75	19	23.75	
Bachelor degree	16	20	20	25	
Master degree and higher	1	1.25	5	6.25	
Income level					
Lower than 10,000,000 Rials	32	40	34	42.50	0/13
1,000,000–15,000,000 Rials	29	36.25	25	31.25	
15,000,000–2,000,000 Rials	16	20	12	15	
Over 2,000,000 Rials	3	3.75	9	11.25	
Spouse’s education					
Lower than diploma	35	43.75	41	51.25	0.09
Associate degree	25	31.2	15	18.75	
Bachelor degree	15	18.75	14	17.5	
Master degree and higher	5	6.25	10	12.5	
Spouse’s job					
Unemployed	0	0	4	5	0.16
Employee	13	16.25	9	11.25	
Others	67	83.75	67	83.75	

The results of the paired t-test showed that the mean scores of preparedness for change, decision-making balance, perceived self-efficacy and change stages before and after the educational intervention in the control group were not significantly different (*P* value > 0.05) (Table 2).

**Table 2:** The mean structures of the stages of change model before and after the intervention in the control group

Variable	Control				*P* value
	Before intervention		After Intervention		
	Standard deviation	Mean	Standard deviation	Mean	
Preparedness for change	3.1	18.43	3.62	18.41	0.56
Decision-making balance	5.95	19.17	3.60	20.36	0.45
Self-efficacy	6.31	17.48	3.56	17.50	0.21
Pre-thinking	4.81	9.15	5.56	9.65	0.72
Thinking	4.50	7.21	4.43	8.01	0.059
Preparation	3.94	8.32	4.21	8.48	0.60
Practice	3.95	8.51	4.84	8.67	0.18
Maintenance	4.41	15.12	3.50	15.77	0.11

The results of the paired t-test indicated that the mean scores of preparedness for change, decision-making balance, perceived self-efficacy, pre-thinking, thinking, preparation, practice and maintenance before and after the educational intervention were significantly different in the experimental group (*P* value < 0.0001) (Table 3).

**Table 3:** The mean structures of the stages of change model before and after the intervention in the experimental group

Variable	Experimental				*P* value
	Before intervention		After Intervention		
	Standard deviation	Mean	Standard deviation	Mean	
Preparedness for change	3.40	19.25	3.58	21.28	< 0.001
Decision-making balance	3.39	19.48	3.85	20.91	< 0.001
Self-efficacy	3.75	19.07	3.62	20.21	< 0.001
Pre-thinking	5.71	9.30	4.74	11.55	< 0.001
Thinking	3.97	8.65	4.29	9.82	< 0.001
Preparation	4.27	10.16	4.17	11.64	< 0.001
Practice	3.84	10.06	4.80	11.66	< 0.001
Maintenance	4.09	17.20	4.36	19.22	< 0.001

The T-test results showed that before the intervention, there was no significant difference between the mean scores of preparedness for change, decision-making balance, perceived self-efficacy, pre-thinking, thinking, preparation, practice, and maintenance in the two groups (*P* value > 0.05) but after the intervention, the mean scores of preparedness for change, decision-making balance, perceived self-efficacy, pre-thinking, thinking, preparation, practice, and maintenance were significantly different in the experimental and control groups (*P* value < 0.05) (Table 4).

**Table 4:** The mean structures of the stages of change model before and after the intervention in the experimental and control groups

Variable	Before intervention		*P* value*	After intervention		*P* value*
	Experiment	Control		Experiment	Control	
	Mean ± standard deviation	Mean ± standard deviation		Mean ± standard deviation	Mean ± standard deviation	
Preparedness for change	19.25 ± 3.40	18.43 ± 3.1	0.06	21.28 ± 3.58	18.41 ± 3.62	0.001*
Decision-making balance	19.48 ± 3.39	19.17 ± 5.95	0.11	20.91 ± 3.85	20.36 ± 3.60	0.04
Self-efficacy	19.07 ± 3.75	17.48 ± 6.31	0.21	20.21 ± 3.62	17.50 ± 3.56	0.001
Pre-thinking	9.30 ± 5.71	9.15 ± 4.81	0.20	11.55 ± 4.74	9.65 ± 5.56	0.001
Thinking	8.65 ± 3.97	7.21 ± 4.50	0.17	9.82 ± 4.29	8.01 ± 4.43	0.001
Preparation	10.16 ± 4.27	8.32 ± 3.94	0.06	11.64 ± 4.17	8.48 ± 4.21	0.001
Practice	10.06 ± 3.84	8.51 ± 3.95	0.07	11.66 ± 4.80	8.67 ± 4.84	0.001
Maintenance	17.20 ± 4.09	15.12 ± 4.41	0.06	19.22 ± 4.36	15.77 ± 3.50	0.001

*P value < 0.001

Table 5 shows the mean and standard deviation of physical activity in the intervention and control groups before and after the intervention. The T-test results showed that after the educational intervention, the mean score of physical activity was significantly higher in the experimental group than in the control group (*P* value < 0.001). The results of the paired t-test showed that the mean score of physical activity before and after the educational intervention was significantly different in the experimental group (*P* value = 0.01), but no significant difference was found in the control group (*P* value = 0.67) (Table 5).

**Table 5:** Mean and standard deviation of physical activity before and after intervention in the experimental and control groups

Group	Before educational intervention (mean ± standard deviation)	After educational intervention (mean ± standard deviation)	*P* value*
Control	341.90 ± 94.80	339.80 ± 90.20	0.67
Intervention	548.30 ± 108.80	726.30 ± 201.60	0.01
*P* value**	< 0.0001	< 0.0001	

*Paired t-test

**Independent t-test

## Discussion

The aim of this study was to investigate the effect of the theory-based educational intervention on the improvement of physical activities of middle-aged women referring to Ahwaz health centers, Iran in 2017. The results of this study showed that the theory-based educational intervention was effective in promoting the physical activities of the middle-aged women. This finding was consistent with the results of various studies that examined the educational intervention to promote the level of physical activities [21–24]. In various studies, some tools including self-efficacy, social support, balance in decision-making, and change strategies were used to change the performance of individuals.

By passing people through different stages of preparation, their physical activities can be promoted. The most applied model in this regard is the theory-based stages of change model [25]. The application and effect of the theory-based educational program for more effective planning of sport interventions have been reported in several studies [26–30]. This model has been derived from basic theories of psychotherapy as the model of behavior change. "Stages of Change" is one of the dimensions of the model that shows the individual's preparedness to change behavior. "Change processes", which form another structure of the theory-based model, show how people change at each stage, and include behavioral and cognitive strategies that individuals use to change their behavior. In their study, Marcus et al., showed that simultaneous use of change processes could be a good guide to do exercise interventions [31]. Education of sports programs based on the theory-based model can increase the transfer of individuals from the early stages of changing sports behaviors to higher stages. Therefore, in order to continue and maintain long-term health behavior, interventions may be necessary to promote physical activities [32]. The results of this study showed that there was no significant difference in the control group in terms of preparedness for change, decision-making balance, self-efficacy, pre-thinking, thinking, preparation, and practice before and after the educational intervention, and the mean scores of all the fields slightly increased after the intervention. These findings were consistent with the results of the studies by Eskandari et al. [33] and Rouholamini et al. [34], who indicated that designing and implementing intervention programs based on change models could improve physical activity behavior in the control group and greatly in the intervention group. The results of the present study showed that there was a significant difference in terms of preparedness for change and balance in decision-making in the intervention group before and after the educational intervention, which was consistent with the results of the study by Solhi et al. [15]. In the present study, there was a significant difference between the mean scores of self-efficacy before and after the intervention in the experimental group, which was consistent with the results of the study by Keshavarz et al. [35]. in their study on self-efficacy of physical activities, Keshavarz et al. showed that after the educational intervention, people’s self-efficacy for doing physical activities significantly increased, and improving the stage of the individuals’ change increased self-efficacy as well [35]. Since one of the most powerful tools for increasing self-efficacy is mastery over behavior, it is possible that self-efficacy changes would occur following successful and active participation of people in physical activities. Analytical findings also suggest that self-efficacy may play the role of an agent for behavioral change, because usually those who show most changes in behavior initially have a higher level of self-efficacy for conducting their behavior [36]. Similarly, the results of this study showed that the components of the educational model did not have a significant difference before the intervention, but their difference was significant after the intervention. These findings were consistent with the results of the study by Kien et al. [26], who showed in their study that educational interventions were effective in promoting the individuals’ physical activities.

One of the strengths of this study was performing community-based educational intervention on women and increasing their physical activity after the intervention.

Another of the strengths of this study was the use of a valid questionnaire to investigate the effect of the educational intervention on physical activities.

In this study, limitations such as a limited period of two months to evaluate the results can be mentioned. Increasing the duration of the intervention and measuring the long-term effects of the intervention could provide more accurate results. Also, the existence of uncontrollable variables including psycho-psychological characteristics, cultural differences, differences in motivation and personal interests of the studied samples that can be gained by their knowledge and performance. Lack of possibility to fully monitor the physical activity of the studied samples are among the weaknesses and limitations of the present study.

## Conclusion

In the present study, the educational intervention based on the stages of change model led to the promotion of physical activity levels in middle-aged women in Ahwaz. Therefore, it is recommended to develop educational programs based on the stages of change model in order to increase the physical activities of middle-aged women. Also, considering that this study is in line with the Health Care System Reform Plan for middle-aged women, its results can be used to better realize the goals of the plan. The results of this research can be used to change the routine methods of educating the help-seekers and those referring to health centers, or to change the educational mission of health care providers.

## Data Availability

The datasets used and/or analyzed during the current study are available from the corresponding author upon reasonable request.

## Abbreviations

WHO: World Health Organization
MET: Metabolic Equivalent of Task

## References

[1] Ferreira M, Sherrington C, Smith K (2012). Physical activity improves strength, balance and endurance in adults aged 40–65 years: a systemic review. J Physiother.

[2] Sylvia LG, Bernstein EE, Hubbard JL, Keating L, Anderson EJ (2014). Practical guide to measuring physical activity. J Acad Nutr Diet.

[3] Bull FC, AlAnsari SS, Biddle S (2020). World Health Organization 2020 guidelines on physical activity and sedentary behavior. Br J Sports Med.

[4] Keshavarz-Mohammadian S, Farmanbar R, Mohtasham-Amiri Z, Atrkar-Roushan Z (2015). Factors associated with physical activity based on the stages of change model among health volunteers in Rasht. Iran J Health Educ Health Promot.

[5] Charkazi A, Nazari N, Samimi A, Koochaki GM, Badeleh MT, Shahnazi H (2013). The relationship between regular physical activity and the stages of change and decisional balance among Golestan University of Medical Sciences’ Students. J Res Dev Nurs Midw.

[6] Tehrani H, Majlessi F, Shojaeizadeh D, Sadeghi R, Kabootarkhani MH (2016). Applying socioecological model to improve women’s physical activity: a randomized control trial. Iran Red Crescent Med J.

[7] Karimzadehshirazi K, Niknami Sh, Faghihzadeh S (2008). Effects of a TTM-based osteoporosis preventive physical activity education, on increasing muscle strength and balance in women aged 40–65. Tehran Hakim Res J.

[8] Anderson E, Durstine JL (2019). Physical activity, exercise, and chronic diseases: a brief review. Sports Med Health Sci.

[9] Kase NG (2009). Impact of hormone therapy for women aged 35 to 65 years, from contraception to hormone replacement. Gend Med.

[10] Qiu M, Sawadogo-Lewis T, Ngale K (2019). Obstacles to advancing women’s health in Mozambique: a qualitative investigation into the perspectives of policy makers. Global Health Res Policy.

[11] Solhi M, Kazemi S S, Haghni H. Relationship between general health and self-efficacy in women referred to health center No.2 in Chaloos (2012). Razi J Med Sci Iran Univ Med Sci. 2013;20(109):72–79

[12] Kolet G, Schofieldet G, Kerse NM, Garrett N (2010). Healthy steps: the effect of primery care physical activity program on quality of in low active older adults. J Sci Med Sport.

[13] Cousins JH, et al. Family versus individualy oriented intervention weight loss in mexican women. 2013, Public Health Rep, pp. 65–71. PMC1403697 1410236

[14] Fallon EA, Hausenblas H, Nigg CR (2005). The transtheoretical model and exercise adherence:examining construct associations in later stages of change. Psychol Sport Exerc.

[15] Solhi M, Ahmadi L, Taghdisi MH, Haghani H (2011). The Effect of Trans Theoritical Model (TTM) on the appropriate physical activities in pregnant women referring to Dehaghan Health Centers. Iran J Educ Med Sci.

[16] Ghahremani L, Niknami SH, Mosavi MT, Heidarnia AR, Shirazi KK, Babaei GH (2008). Transtheoretical model-based (TTM) interventions to improve physical activities in elderly men. Armaghane Danesh Bimonthly J.

[17] Galway LP, Bell N, AlShatari SAE (2012). A two-stage cluster sampling method using gridded population data, a GIS, and Google EarthTM imagery in a population-based mortality survey in Iraq. Int J Health Geograph.

[18] Taherdoost H (2016). Sampling methods in research methodology; how to choose a sampling technique for research. Int J Acad Res Manag (IJARM).

[19] Turner AG, Magnani RJ, Shuaib MA (1996). Not quite as quick but much cleaner alternative to the expanded programme on immunization (EPI) cluster survey design. Int J Epidemiol.

[20] Working group for Mortality Estimation in Emergencies (2007). Wanted: studies on mortality estimation methods for humanitarian emergencies, suggestions for future research. Emerg Themes Epidemiol.

[21] Vahedian Shahroodi M, Tavakoly Sany SB, Hosseini Khaboshan Z, Esmaeily H, Jafari A, Tajfard M. Effect of a theory-based educational intervention for enhancing nutrition and physical activity among Iranian women: a randomised control trial. Public Health Nutrition. Cambridge University Press; 2021;1–12. 10.1017/S1368980021002664 PMC11148578 34420543

[22] Darabi F, Kaveh MH, Majlessi F, Farahani FKA, Yaseri M, Shojaeizadeh D (2017). Effect of theory-based intervention to promote physical activity among adolescent girls: a randomized control trial. Electron Physician.

[23] Zare F, Aghamolaei T, Zare M, Ghanbarnejad A (2016). The effect of educational intervention based on the transtheoretical model on stages of change of physical activity in a sample of employees in Iran. Health Scope..

[24] Taghipour A, Shahroudi MV, Tabesh H, Safari-Moradabadi A, Alipour AM (2019). The effect of educational intervention based on the theory of planned behavior and stages of change construct on women's physical activity. J Educ Health Promot.

[25] Hashemzadeh M, Rahimi A, Zare-Farashbandi F, Alavi-Naeini AM, Daei A (2019). Transtheoretical model of health behavioral change: a systematic review. Iran J Nurs Midwifery Res.

[26] Kien TL, Yee CK, Wan NA, Youngho K, Garry K (2018). Application of transtheoretical model on behavioral changes, and amount of physical activity Among University’s Students. Front Psychol.

[27] Yang HJ, Chen KM, Chen MD, Wu HC, Chang WJ, Wang YC (2015). Applying the transtheoretical model to promote functional fitness of community older adults participating in elastic band exercises. J Adv Nurs.

[28] Elezim A, Elezi G, Gontarev S, Georgiev G (2020). Application of the Transtheoretical Model (TTM) to exercise behaviour among Macedonian college students. J Human Sport Exerc.

[29] Moosavi S, Farmanbar R, Fatemi S, Yazdanipour MA (2017). The Effect of a TTM-based intervention on level of physical activity in ICU nurses. Iran Red Crescent Med J.

[30] Han H, Gabriel KP, Kohl HW (2017). Application of the transtheoretical model to sedentary behaviors and its association with physical activity status. PLoS ONE.

[31] Marcus BH, Rakowskiw W, Rossi JS (1992). Assessing motivational readiness and decision making for exercise. Health Psycho.

[32] Khosronia L, Jafari F, Hajimiri K (2020). Effect of education based on trans-theoretical model on physical activity of reproductive aged women referred to health centers in Zanjan. J Hum Environ Health Promot.

[33] Eskandari N, Araban M, Saki MA (2015). Promotion of physical activity of women referring to health centers using the model of metatheory. Q J Health Educ Promot.

[34] Rouholamini S, Gheibizadeh M, Maraghi E, Jahanshahi A (2020). The effects of a training program based on the health promotion model on physical activity in women with type 2 diabetes: a randomized controlled clinical trial. Iran J Nurs Midwifery Res.

[35] Keshavarz Mohammadian S, Farmanbar R, Mohtasham Amiri Z, Atrkar RZ (2019). Effect of “stage of change model” on the promotion of physical activity of health volunteers in Rasht. J Guilan Uni Med Sci.

[36] Pirzadeh A, Mostafavi F, Ghofranipour F, Feizi A (2015). Applying transtheoretical model to promote physical activities among women. Iran J Psychiatry Behav Sci..

